# Characterization of Muscle Weakness Due to Myasthenia Gravis Using Shear Wave Elastography

**DOI:** 10.3390/diagnostics13061108

**Published:** 2023-03-15

**Authors:** Manuela Zimmer, Benedict Kleiser, Justus Marquetand, Filiz Ates

**Affiliations:** 1 Institute of Structural Mechanics and Dynamics in Aerospace Engineering, University of Stuttgart, Pfaffenwaldring 27, 70569 Stuttgart, Germany; 2 Department of Epileptology, Hertie-Institute for Clinical Brain Research, University of Tübingen, Hoppe-Seyler-Str. 3, 72076 Tübingen, Germany; 3 Department of Neural Dynamics and Magnetoencephalography, Hertie-Institute for Clinical Brain Research, University of Tübingen, Otfried-Müller-Str. 25, 72076 Tübingen, Germany; 4 MEG-Center, University of Tübingen, Otfried-Müller-Str. 47, 72076 Tübingen, Germany

**Keywords:** myasthenia gravis, shear wave elastography, surface electromyography, skeletal muscle mechanics, muscle stiffness, muscle weakness, biceps brachii

## Abstract

Myasthenia gravis (MG) is often accompanied with muscle weakness; however, little is known about mechanical adaptions of the affected muscles. As the latter can be assessed using ultrasound shear wave elastography (SWE), this study characterizes the biceps brachii muscle of 11 patients with MG and compares them with that of 14 healthy volunteers. Simultaneous SWE, elbow torque and surface electromyography measurements were performed during rest, maximal voluntary contraction (MVC) and submaximal isometric contractions (up to 25%, 50% and 75% MVC) at different elbow angles from flexion to extension. We found that, with increasing elbow angle, maximum elbow torque decreased (*p* < 0.001), whereas muscle stiffness increased during rest (*p* = 0.001), MVC (*p* = 0.004) and submaximal contractions (*p* < 0.001). Muscle stiffness increased with increasing contraction intensities during submaximal contractions (*p* < 0.001). In comparison to the healthy cohort, muscle stiffness of MG patients was 2.1 times higher at rest (*p* < 0.001) but 8.93% lower in active state (75% MVC, *p* = 0.044). We conclude that (i) increased muscle stiffness shown by SWE during rest might be an indicator of MG, (ii) SWE reflects muscle weakness and (iii) SWE can be used to characterize MG muscle.

## 1. Introduction

Characterized by muscle weakness, myasthenia gravis (MG) is a pathophysiological disorder of the neuromuscular junction, mostly due to an autoimmune etiology. With a worldwide prevalence of 12.4 people per 100,000 population [1], it is one of the most common neuromuscular diseases [2]. Characteristically, muscle weakness increases after physical activity [3,4] and both central [5] as well as peripheral fatigue [6] have been reported for MG patients. Depending on the affected muscles, MG is classified as ocular MG if eye muscles are affected, oropharyngeal MG if difficulties exist in articulation, chewing or swallowing, or generalized MG if limb weakness is present that can be accompanied by ocular and oropharyngeal problems [3]. There are various scales to evaluate muscle weakness [7], such as the quantitative MG score originally proposed by Besinger et al. in 1983 [8]. It is one of the most commonly used scores and is recommended by the Myasthenia Gravis Foundation of America [9]. Via the Besinger Score, eight typical symptoms regarding ocular, oropharyngeal and generalized involvement are rated with points between 0 (normal) and 3 (severe) [8,9]. Even though the score describing the overall condition of patients is helpful in the clinic to monitor the disease and test therapy responses, it is only a rough classification and the outcome is dependent on the motivation of the patients. Skeletal muscle weakness is evaluated by a duration of the tests where patients are asked to hold their arms, legs or head in a certain position as long as possible. It is apparent that such tests alone cannot quantify muscle weakness thoroughly. Consequently, a diagnostic tool for the daily clinical routine is needed that not only measures muscle strength or weakness, but also has the potential to quantify muscle characteristics objectively.

Ultrasound shear wave elastography (SWE) could be such a diagnostic tool. As a non-invasive method, SWE quantifies local muscle mechanical properties such as muscle stiffness—a muscle characteristic that has not been comprehensively studied in MG. With a specially designed ultrasound probe, shear waves are induced that propagate perpendicularly to the main transducer transmission direction. SWE performs real-time tracking of the shear waves from the B-mode ultrasound images and calculates a 2D map of the shear wave speed. Assuming transverse isotropic and linear elastic material characteristics of muscle tissue, the shear wave speed is quadratically related to the shear elastic modulus. A previous study validated that the shear wave speed measured in longitudinal muscle orientation provides a measure of local muscle stiffness [10]. Furthermore, SWE was shown to represent both passive [11,12] and active muscle mechanics in vivo [13,14,15]. In particular, these studies revealed the potential of SWE in muscle characterization by demonstrating that shear wave speed increases with increasing muscle length in passive condition (i.e., in a relaxed state without voluntary contraction) [11,12,13] and that shear wave speed increases with contraction intensity during isometric contractions [13,14,15]. A recent study [16] has argued that these relationships were established assuming unloaded condition for muscles and the increase in shear wave speed is not only attributed to an increase in muscle stiffness (shear elastic modulus) but may also be more dominantly due to an increase in tensile loading conditions of the muscle. Moreover, Wang et al. highlighted the importance of taking viscoelastic muscle behavior into account when interpreting SWE results [17]. Despite such challenges in relating the measured shear wave speed to muscle stiffness, previous studies denoted the promising clinical use of SWE in muscle diagnostics and therapeutic monitoring, e.g., for patients with myositis, stroke, cerebral palsy or multiple sclerosis [18]. SWE was claimed to be a biomarker in myotonia as the muscle relaxing time estimated using SWE was similar to those obtained with mechanical dynamometers [19]. Increased shear wave speed under passive conditions of the biceps brachii muscle (BB) was found in patients with Parkinson’s Disease [20] and it was shown that SWE output correlates with joint rigidity [21]. Therefore, SWE might pose a new non-invasive application for various diseases affecting the neuromuscular system.

From a biomechanical perspective, structural and hence mechanical adaptations of muscles might occur due to MG as a result of altered synaptic transmission. Thus, SWE can be used to investigate the mechanical changes reflected by muscle stiffness that can arise from the underlying muscle weakness. Consequently, the purpose of this study was to characterize the mechanical properties of muscles affected by MG described by their local muscle stiffness in both passive (i.e., rest) and active conditions (i.e., contraction with different force levels). By comparing the data with the previous work on healthy muscles using the same experimental setup [13], we aimed to determine the mechanical adaptation of muscles in MG. Therefore, we hypothesized that (i) passive muscle stiffness increases with increasing muscle length, muscle stiffness in active state (ii) increases with increasing contraction intensity, (iii) shows a length-dependent characteristic for MG patients, and (iv) the muscle weakness associated with MG leads to alterations in both passive and active muscle mechanics compared to healthy muscle.

## 2. Materials and Methods

### 2.1. Participants

Eleven patients (4 males; 1 left-handed; age: 47.64 ± 15.74 years; body mass index (BMI): 26.42 ± 4.19 kg/m^2^; body weight: 74.73 ± 13.19 kg; body height: 168.18 ± 9.51 cm; all data given as mean ± standard deviation) diagnosed with MG participated in the present study. Data collected from 14 participants (7 males; all right-handed; age: 28.07 ± 5.06 years; BMI: 24.24 ± 3.78 kg/m^2^; body weight: 77.21 ± 17.37 kg; body height: 177.71 ± 7.53 cm) in identical conditions were used for comparing the present results with a healthy population (a comprehensive characterization of the healthy cohort can be found elsewhere [13]). Patients with different characteristics regarding antibody specificity, thymus pathology, treatment options and muscle weakness were included (Table 1). A severe Besinger arm holding test score (>2 points) was chosen as an exclusion criterion to ensure that patients can perform the experimental protocol without becoming exhausted. All patients were taking their regular medication to reduce their daily symptoms including muscle weakness. Patients were asked to refrain from any exercise 24 h prior to the study to prevent any possible side effects and the time of the study was chosen to be as close as possible before the daily intake of medication.

### 2.2. Anthropometric Data Collection

At rest, the perimeter of the upper arm was measured. The cross-sectional area (CSA) of the BB muscle was measured using B-mode ultrasound images (AixPlorer Mach30, MSK preset, linear transducer L18-5, 5–18 MHz, 50 mm wide, Supersonic Imagine, Aix-en-Provence, France) and the length of the BB was quantified by identifying the proximal and distal ends of the muscle for each elbow angle tested (60°, 90°, 120°, 150° and 180°).

### 2.3. Experimental Measurements

**Figure 1:**
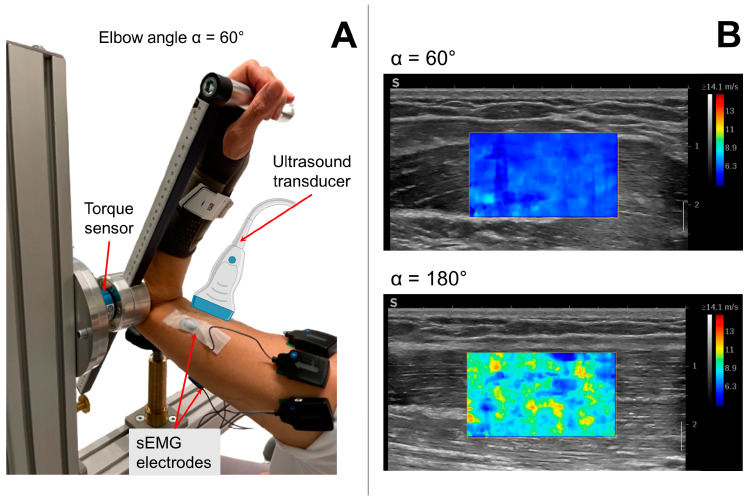
(**A**) Experimental setup showing a patient’s left arm placed in the custom-made apparatus. The elbow joint axis is aligned with the rotational axis of the torque sensor. Surface electromyography (sEMG) electrodes are positioned at the biceps brachii (BB) and triceps brachii muscle. The ultrasound transducer is placed on top of the BB muscle belly and aligned with muscle fiber direction. (**B**) Exemplary ultrasound frames including shear wave propagation speed color overlays during 50% isometric ramp contractions for two elbow angles (60°, 180°) studied.

A custom-made apparatus that allows fixing elbow angles at designated positions (Figure 1A) was used for isometric elbow flexion and extension torque measurements. Five elbow angles from flexed position at 60° to fully extended position at 180° were studied with 30° intervals.

Electrical activity of BB and triceps brachii muscle (TB) was measured with dry-surface electrodes (10 mm inter-electrode spacing) of the Trigno Wireless Biofeedback System (Delsys Europe, Greater Manchester, UK). Electrode location followed the SENIAM recommendations [22] with two electrodes placed in the long and lateral head of the TB and two electrodes placed on the BB slightly medial and lateral to the central position to allow simultaneous SWE measurements.

Shear wave propagation velocity was measured using an ultrasound device providing SWE (AixPlorer Mach30, MSK preset; Supersonic Imagine, Aix-en-Provence, France) with a linear transducer (5–18 MHz, 50 mm wide; L18-5; Supersonic Imagine, Aix-en-Provence, France). The transducer was aligned with the muscle fiber direction and placed in the middle of the BB belly in between the sEMG electrodes (Figure 1A). The shear elastic modulus G that is associated with shear waves traveling parallel to the muscle fibers with polarization perpendicular to them was calculated from the shear wave velocity assuming transverse isotropic, lossless linear elastic material characteristics of the muscle in unloaded condition as follows:

G = ρ·v_s_^2^, (1)

where ρ is the mass density (with ρ_muscle_ ≈ 1.0 kg/m^3^). A more detailed mathematical description and derivation of Equation (1) can be found elsewhere (e.g., [16]).

The maps of the shear wave velocity were recorded at 0.8–1.8 Hz depending on the size and position of the region of interest (ROI). The maximum shear wave speed (or shear elastic modulus) provided by the system was 14.1 m/s (or 200 kPa). sEMG and torque signals were recorded via a data acquisition system (cDAQ-9174, National Instruments, Austin, TX, USA) and sampled with 2 kHz. A custom MATLAB (The MathWorks, Inc., Natick, MA, USA) script was used to control the data acquisition system, visualization and recording of data and allowed one to start and stop the recordings of the ultrasound system synchronously.

### 2.4. Experimental Protocol

Simultaneous measurements of elbow torque, sEMG and ultrasound videos (B-Mode and shear wave velocity maps) were performed on the patient’s dominant arm. The trials included 5 s isometric maximum voluntary contractions (MVCs) for both elbow flexion and extension, 5 s resting trials, where patients were asked to relax their muscle as much as possible, and isometric ramp contractions (for elbow flexion only). The ramp contractions consisted of (I) 3 s rest, (II) a ramp up to 25%, 50% or 75% MVC torque (with 6.25% per second slope), (III) 5 s constant level, (IV) a ramp down to 0% MVC torque (with 6.25% per second slope) and (V) 3 s rest. Patients were given visual feedback to perform the ramp contractions by following a given line with a marker representing their applied elbow torque. The experiments started with a warm-up protocol. It consisted of one elbow flexion MVC and ramp contractions up to 25% and 50% MVC torque at two joint positions (120° and 180° elbow angles). The following experimental procedures are described in Table 2 and steps 2–6 were repeated for each elbow angle studied (60°, 90°, 120°, 150° and 180°).

### 2.5. Data Analysis

The maximum elbow torque was calculated for each elbow angle from the MVC trials. Normalized MVC elbow flexion torque was calculated by normalizing the torque data to each patient’s maximum value. For the submaximal ramp contractions, the normalized mean torque and the mean absolute percentage deviation (MAPD) of applied torque from the given, ideal ramp were calculated for the middle three seconds of section III. Trials above 10% MAPD were excluded from further analysis (including sEMG and SWE) to minimize the inclusion of performance errors.

All sEMG recordings were inspected visually for measurement errors (e.g., electrode attachment became loose, operator touched the electrode). The sEMG data were filtered (fourth order Butterworth filter, 20–350 Hz bandwidth) and full-wave rectification was applied. The root mean square moving average with 250 ms windows was calculated and is used as a measure of the sEMG amplitude throughout this manuscript. The signals collected from the two BB electrodes and two TB electrodes were averaged and used as representatives of sEMG of BB and TB, respectively. For all trials, the sEMG amplitude was averaged over the middle 3 s of the measurements (middle 3 s of section III for ramp trials). The MVC sEMG amplitude was used for the normalization of the resting trials for each elbow angle studied. sEMG amplitudes of ramp contraction trials were normalized to the average amplitude during the constant level (section III) of the 75% ramps for both BB and TB.

Color analysis of the SWE videos (i.e., decoding the color information from the 2D color map to numerical values) was performed for each trial individually. Inside the color map, an ROI was defined manually covering only muscle fibers and the average shear elastic modulus was calculated. The recordings were analyzed frame by frame according to the SWE sampling rate. If more than 25% of the pixels inside the ROI were missing, no result was assigned to that frame. For analysis of rest and MVC trials, the mean shear elastic modulus was calculated from all available frames. Ramp contraction recordings were resampled to 1 Hz from their original SWE sampling rate and mean shear elastic modulus was calculated from the middle three seconds of the constant level (section III).

During rest, the trials with sEMG amplitude of the BB below 3% were considered as representing the passive state of muscles and passive shear elastic modulus was evaluated only for the trials that met this exclusion criterion.

During MVC and ramp contractions, the active shear elastic modulus is calculated by subtracting the shear elastic modulus during rest from the measured (i.e., total) shear elastic modulus.

### 2.6. Statistics

To determine the effect of elbow angle, muscle contraction intensity level and disease on the measurands, analysis of variation (ANOVA) with a significance level of α = 0.05 was performed. Pairwise comparison tests were performed with the Bonferroni method as post-hoc analyses. Pearson correlation tests with a significance level of α = 0.05 were performed to determine the linear relationship between (i) the maximum elbow flexion torque production and BB muscle physiological, electrical and mechanical properties measured as well as (ii) the electrical activity of the BB at rest and patient characteristics.

## 3. Results

### 3.1. Anthropometrics

All data in the following sections are presented as mean ± standard deviation. The average upper arm circumference was 31.64 ± 2.80 cm. Elbow angle had a significant effect on BB length (*p* < 0.001) but not on BB CSA (*p* = 0.970). Post-hoc analysis showed significant differences for muscle lengths between 60° and 120° (*p* = 0.001), 150° (*p* < 0.001), 180° (*p* < 0.001), 90° and 150° (*p* < 0.001), 180° (*p* < 0.001) as well as 120° and 150° (*p* = 0.047), 180° (*p* < 0.001) elbow angles. The BB lengths were 11.71 ± 1.47 cm, 13.31 ± 1.43 cm, 14.42 ± 1.56 cm, 16.35 ± 1.61 cm and 17.75 ± 1.56 cm (for 60°, 90°, 120°, 150° and 180° elbow angle, respectively). The average CSA over all elbow angles was 8.25 ± 3.10 cm^2^.

### 3.2. Active Muscle Characteristics

#### 3.2.1. MVC

**Figure 2:**
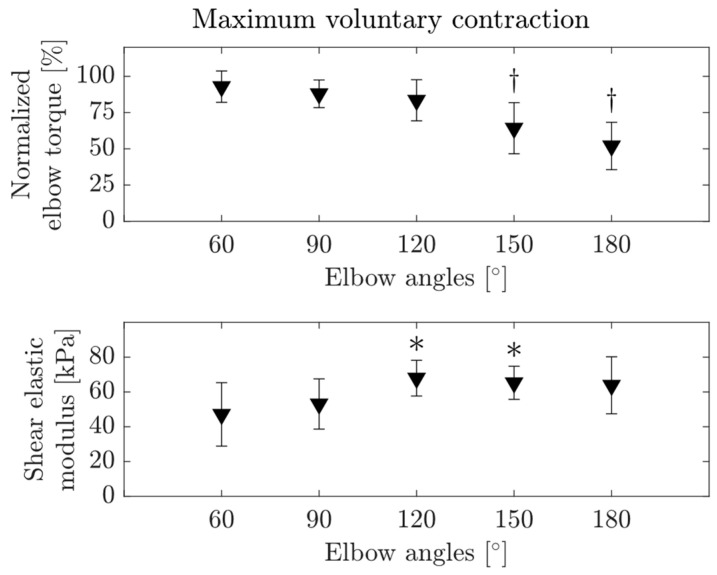
Average normalized elbow flexion torque and total shear elastic modulus of the biceps brachii muscle during maximum voluntary contractions at the five elbow angles studied (60°, 90°, 120°, 150° and 180°). Error bars visualize the standard deviation. The cross and star signs indicate that the measured value is significantly different from the normalized elbow torque at 60°, 90°, 120° and the shear elastic modulus measured at 60° elbow angle (*p* < 0.05), respectively.

The maximum elbow flexion torque ranging from 4.60 Nm to 76.02 Nm showed substantial inter-individual variance (Table 3). The elbow angle did not have a significant effect on the absolute MVC torque (*p* = 0.238); however, normalized flexion torque during MVC decreased with increasing elbow angle (*p* < 0.001, Figure 2, upper panel) with significant differences between 60° and 150° (*p* < 0.001), 180° (*p* < 0.001) and 90° and 150° (*p* = 0.004), 180° (*p* < 0.001) and 120° and 150° (*p* = 0.035), 180° (*p* < 0.001) elbow angles. Maximum average normalized torque was 92.88% at 60° elbow angle and it decreased to 51.97% at 180° elbow angle.

While sEMG amplitudes of the BB (*p* = 0.952) and TB (*p* = 0.901) and active shear elastic modulus (*p* = 0.791) did not change with the elbow angle, a significant effect of elbow angle was found for the total shear elastic modulus (*p* = 0.004, Figure 2, lower panel). Post-hoc tests located the differences between 60° and 120° (*p* = 0.013) and 60° and 150° (*p* = 0.041).

#### 3.2.2. Submaximal Ramp Contractions

Out of 165 ramp trials, 10 (7 trials of the 75% ramps and 3 trials of the 50% ramps) were excluded due to subjects being unable to maintain the desired torque level (MAPD > 10%). The average MAPD (over elbow angles) calculated from the middle 3 s of section III was 1.84%, 2.59% and 5.09% for 25%, 50% and 75% ramps, respectively.

**Figure 3:**
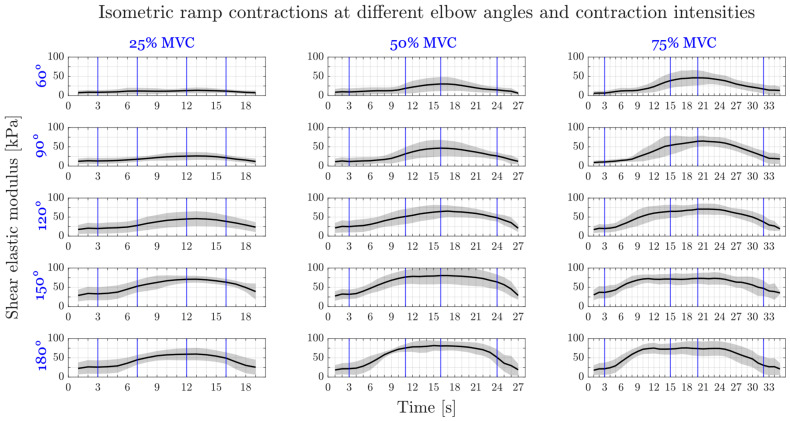
Average total shear elastic modulus of the biceps brachii muscle during elbow flexion isometric ramp contraction trials with standard deviation (in light grey shades). Columns show the three contraction intensities with a constant level up to 25%, 50% and 75% of maximum voluntary (MVC) elbow torque, rows show the five elbow angles studied (60°, 90°, 120°, 150° and 180°). Vertical blue lines indicate the different sections of the trapezoid line to be followed.

The shear elastic modulus during ramp contractions followed the increase and decrease in elbow torque (Figure 3). Two-way ANOVA showed significant effects of elbow angle and contraction intensity on total shear elastic modulus measured at the constant level (section III, *p* < 0.001 for both). Post-hoc tests located the significant differences in total shear elastic modulus between 60° and 90° (*p* = 0.005), 120°, 150°, 180° (*p* < 0.001 for the remaining angles), between 90° and 120° (*p* = 0.009), 150°, 180° (*p* < 0.001 for the remaining angles), and between 120° and 150° (*p* = 0.001), 180° (*p* = 0.017). Furthermore, the total shear elastic modulus at 25% MVC torque was significantly different than at 50% (*p* < 0.001) and 75% (*p* < 0.001) MVC torque.

Elbow angle and contraction intensity had similar effects on active shear elastic modulus: Significant differences were found (i) between 60° and 120° (*p* = 0.002), 150°, 180° (*p* < 0.001 for both), 90° and 150° (*p* = 0.009), 180° (*p* = 0.001), and 120° and 180° (*p* = 0.004) and (ii) between 25% MVC torque and 50% (*p* < 0.001) and 75% (*p* < 0.001) MVC torque.

Only contraction intensity had a significant effect on sEMG amplitude of BB (*p* = 0.019 for elbow angle, *p* < 0.001 for contraction intensity) and TB (*p* = 0.109 for elbow angle, *p* < 0.001 for contraction intensity). sEMG amplitude differed between 25% and 50%, 75%, and also between 50% and 75% for both BB and TB (*p* < 0.001 for all).

### 3.3. Passive Muscle Characteristics

**Figure 4:**
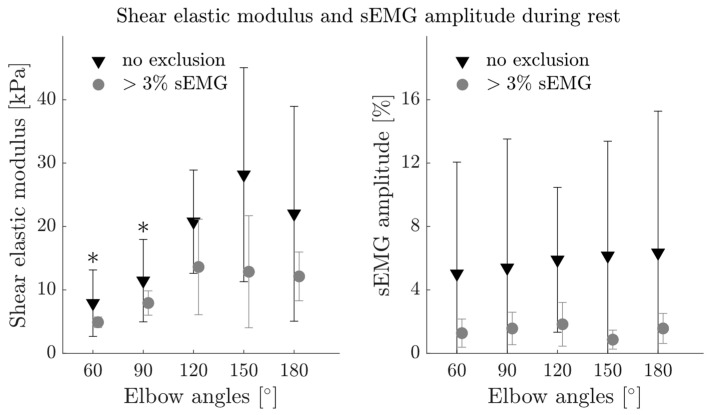
Average shear elastic modulus of the biceps brachii muscle (BB) and sEMG amplitude of the BB during rest (no exclusion, black triangles) and with the exclusion of the sEMG amplitude > 3% (grey circles). Error bars visualize the standard deviation. * Significant different to 150° elbow angle (*p* < 0.05).

During rest, elbow angle had a significant effect on shear elastic modulus (*p* = 0.001) (Figure 4). Post-hoc tests located the differences between 60° and 150° (*p* = 0.002) and 90° and 150° (*p* = 0.018). sEMG amplitude of the BB was greater than 3% for 52.73% of the resting trials. Excluding trials during rest with an sEMG amplitude greater than 3% resulted in a total of 5, 5, 3, 5 and 8 patients for 60°, 90°, 120°, 150° and 180° elbow angles, respectively. After the exclusion, elbow angle did not show significant effects on passive shear elastic modulus (*p* = 0.069). Mean sEMG amplitude of BB before and after exclusion was 5.76% ± 0.55% and 1.42% ± 0.37%, respectively (Figure 4).

### 3.4. Associations between the Physiological, Mechanical and Electrical Properties Tested

The point-biserial correlation test revealed significantly higher BB CSA (*p* = 0.007, *p* = 0.008, *p* = 0.008, *p* = 0.007, *p* = 0.017 for 60°–180° elbow angles, respectively) as well as MVC flexion (*p* < 0.001 for all angles) and extension (*p* < 0.001) torque for male patients compared to females. MVC torque measured at some elbow angles was found to be positively correlated with BB CSA and the total shear elastic modulus at MVC and negatively correlated with the shear elastic modulus and sEMG at rest (Table 4). Similarly, sEMG at rest was found to be positively correlated with the shear elastic modulus at rest (Table 5). On the other hand, sEMG at rest did not show strong relations with the patient characteristics: It was only correlated with age at 120° and BMI at 60° elbow angles (Table 5).

### 3.5. Comparisons between MG Patients and Healthy Group

Compared to healthy participants [13], the shear elastic modulus of BB during rest was 2.1 times higher for MG patients (*p* < 0.001) and elbow angle had a significant effect (*p* < 0.001) without interaction (*p* > 0.05).

**Figure 5:**
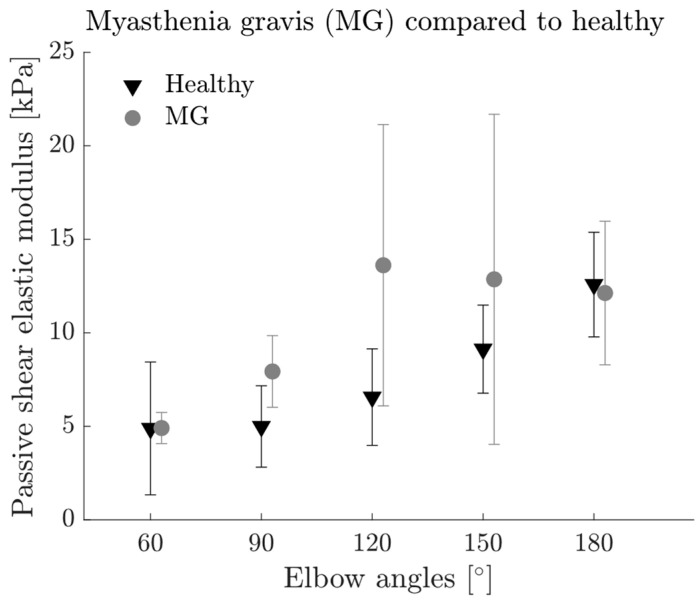
Average passive shear elastic modulus of the biceps brachii muscle of myasthenia gravis (MG) patients (grey circles) compared to healthy population (black triangles) reported previously [13] at passive state for the five elbow angles studied (60°, 90°, 120°, 150° and 180°). Error bars visualize the standard deviation. Two-way ANOVA revealed significant differences between the groups (*p* = 0.002) and elbow angles (*p* < 0.001).

The passive shear elastic modulus (meeting the exclusion criterion of <3% sEMG amplitude) was on average 40.83% higher for MG patients compared to the healthy group (*p* = 0.002, Figure 5). Elbow angle had a significant effect on passive shear elastic modulus (*p* < 0.001) and no interactions were found between the factors (*p* > 0.05).

Maximum flexion torque was on average 26.46% lower in MG patients (*p* < 0.001) and it decreased with increasing elbow angle (*p* < 0.001) with no significant interaction (*p* > 0.05). Maximum extension torque was 29.78% lower (*p* = 0.035) for MG patients. The total shear elastic modulus during MVC was not significantly affected by disease (*p* = 0.405) but the active shear elastic modulus was 15.18% less for the MG patients (*p* = 0.013). Elbow angle did not have a significant effect on total (*p* = 0.032) or active shear elastic modulus (*p* = 0.708) during MVC.

**Figure 6:**
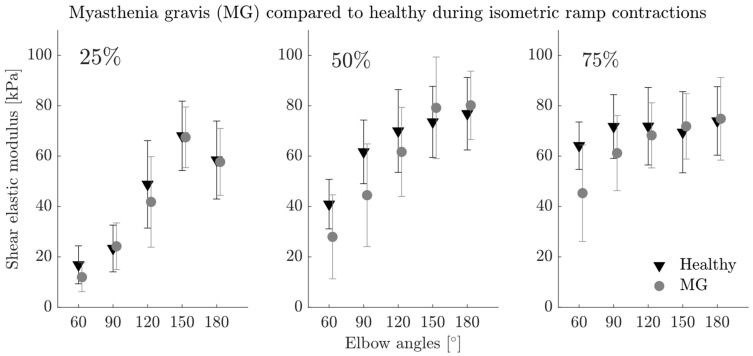
Average total shear elastic modulus of the biceps brachii muscle of myasthenia gravis (MG) patients (grey circles) compared to the healthy group (black triangles) reported previously [13] at 25%, 50% and 75% of maximum voluntary contraction (MVC) torque for the five elbow angles studied (60°, 90°, 120°, 150° and 180°). Error bars visualize the standard deviation. Two-way ANOVA showed significant differences between the groups at 75% MVC (*p* = 0.044).

During submaximal ramp contractions, we found significant differences of the total shear elastic modulus (two-way ANOVA for each contraction intensity) without interaction (*p* > 0.05) between MG and healthy groups at 75% (*p* = 0.044) but not for 25% (*p* = 0.312) or 50% (*p* = 0.051) MVC torque (Figure 6). The shear elastic modulus was on average 8.93% lower for MG patients at 75% MVC torque. The elbow angle had a significant effect on shear elastic modulus for all contraction intensities (*p* < 0.001 for all).

The active shear elastic modulus was significantly lower for MG patients compared to the healthy group for all contraction intensities (*p* < 0.001 for 25%, 50% and 75% MVC torque). It was 38.99%, 28.08% and 21.99% lower at 25%, 50% and 75% MVC torque, respectively. Elbow angle had a significant effect for 25% and 50% MVC torque (*p* < 0.001 for both) but not for 75% (*p* = 0.217).

## 4. Discussion

This is the first study that comprehensively investigates the biomechanical properties of diseased muscle in MG using simultaneous SWE, sEMG and joint torque measurements. Analyzing the mechanical and electrical properties of BB at different joint positions at different contraction intensities from rest to MVC, we can interpret and relate changes in muscle stiffness with muscle length and resulting joint torque production. Comparing the findings with the healthy cohort [13] gives valuable insights in altered mechanical properties of muscles affected by MG and highlights the potential use of SWE as a diagnostic tool. The present results support the hypotheses that SWE can detect changes in muscle stiffness imposed by joint position, contraction intensity and MG.

### 4.1. Towards In Vivo Muscle Force–Length Characteristics

We found that the maximum elbow torque is produced at 60° elbow angle (the most flexed joint position tested) for MG patients. Earlier studies on BB muscle force estimation showed that the maximum torque was generated around 90° elbow angle [23,24,25]. This might be due to the probable differences between the experimental setups (e.g., shoulder or wrist positioning) since present findings are in accordance with our previous data collected from healthy participants at identical conditions [13].

From single muscle fiber experiments, the force–length relationship of muscle fiber is well described by an increase of force with increasing fiber length up to a plateau phase indicating optimal length and decreasing force with further lengthening depending on the number of myofilament binding sites [26]. Even though there are additional determinants of muscle force production such as arrangement of sarcomeres, muscle architecture, geometry and connective tissue involvement, in muscle level, force–length characteristics follow a similar pattern [27,28]. Transferring this characteristic to the in vivo situation, in particular, during voluntary contraction is more complicated as even more factors (e.g., contribution of synergistic and antagonistic muscles [29,30], force transmission through passive structures) influences the resulting joint torque that can be measured non-invasively. From the elbow torque measurements and the decline in maximum torque production with lengthening of elbow flexors, we might infer that the BB and/or other elbow flexor muscles are operating in the descending limb of their force–length characteristics (from elbow flexion to extension in vivo) for both MG patients and healthy adults. However, one should keep in mind that the resulting joint torque is not necessarily a valid representation of each muscle’s individual force production.

On the other hand, muscle shear elastic modulus deduced from SWE can be used to describe in vivo muscle characteristics in a more meaningful way compared to joint torque measurements as it characterizes the local muscle properties of an individual muscle. Relating the measured shear elastic modulus to the muscle’s force production is not straightforward either. Besides the aforementioned challenges, it should be noted that the measured muscle stiffness reflects both passive and active muscle states combined. Previous in vivo studies of skeletal muscles found an increasing shear wave speed with increasing muscle length imposed by joint position during rest [12,13,17,31] and, in active state, increased shear wave speed with increasing contraction intensity reflected by the resulting joint torque [13,14,15,17]. However, the increase in shear wave speed may not only be attributed to the changes in muscle stiffness but also to an increase in tensile loading [16]. In particular during active muscle contractions, the impacts of active force production might be much higher. Based on their experiments on cat soleus muscle, Bernabei et al. speculated that the net change in shear wave speed might be ascribed in nearly equal proportions to force-dependent changes in muscle stiffness and to changes in muscle force [32]. If in the future those factors can be reliably separated and quantified from the measured shear wave speed, SWE has great potential to be further developed as an index of muscle force. Thereby SWE can be used as a non-invasive quantitative measure of muscle weakness in MG, improving diagnostics, monitoring and making treatment decisions.

For MG patients during MVC, we found a decrease in maximum elbow torque but an increase in BB stiffness with increasing muscle length. Thus, from the decreasing elbow torque with increasing muscle length, we expect a decreasing muscle stiffness and from the passive state of the muscle during rest, we expect an increasing muscle stiffness with increasing muscle length. From the present results, we conclude that the increase in passive muscle stiffness induced by the lengthening of the muscle is greater than the decrease in muscle stiffness related to the torque production which might explain the increase in BB shear elastic modulus during MVC at extended joint positions. The previously reported muscle stiffness characteristics of the healthy group did not show a muscle length effect during MVC [13]. Compared to the healthy group, passive muscle stiffness might have a bigger influence on the total shear elastic modulus for MG patients as the active force production capacity is reduced (reflected by the reduced maximum elbow torque). To fully draw this conclusion, we calculated the active contribution of muscle stiffness by subtracting the stiffness measured during rest from the total stiffness and the effect of elbow angle disappeared.

However, the muscle stiffness measured during rest is not necessarily equivalent to the passive muscle stiffness. In previous studies, the muscle was considered to be in a passive state if the sEMG amplitude was below a certain threshold [33], although there is no common standard and a wide range of thresholds from 1% up to 10% of normalized sEMG amplitude is used [34]. Considering that more than half of the patients had muscle activity greater than 3%, the shear elastic modulus measured during rest does not describe the passive muscle state of MG patients. Nonetheless, we assumed the influence of the low-level activity during rest on the shear elastic modulus measured during MVC not to be prominent. Therefore, the calculation of the active shear elastic modulus during MVC is reasonable and the fact that the change in active stiffness with muscle length during MVC found for MG patients is similar to the healthy participants [13] supports that.

Calculated from less than half of the patients (with <3% sEMG activity during rest), the passive shear elastic modulus not changing with increasing muscle length does not support our hypothesis or align with the findings from the healthy cohort [13]. Interestingly, the shear elastic modulus at resting state showed an increase with muscle length (Figure 4, left panel), while muscle activity did not differ between the joint positions (Figure 4, right panel). Therefore, the increase in shear elastic modulus cannot be explained by changes in muscle activity. We conclude that the small sample size for the passive shear elastic modulus is responsible for the lack of significant effect.

The findings during submaximal ramp contractions, which support our hypotheses, suggested that muscle stiffness deduced from SWE measurements increases with increasing torque production and differences imposed by muscle length can be detected using SWE. The effect of contraction intensity was significant between 25% and the higher levels (50% and 75% MVC torque) but not between 50% and 75%. Presumably, the maximum BB force production capability reflected by its stiffness is reached before maximum elbow flexion torque. Therefore, it should be the contribution of other elbow muscles causing a further increase in the elbow torque. This also explains the unexpected finding that during MVC the shear elastic modulus does not decrease even though the maximum elbow torque decreases at extended joint positions. Moreover, the muscle length effects during submaximal contractions are more prominent at 25% and 50% MVC torque (Figure 6) compared to the high contraction intensities (75% MVC torque and MVC). Although the maximum elbow torque production decreases with increasing elbow angle, the shear elastic modulus increases. Following our assumption that maximum BB force production is reached before maximum elbow flexion torque, the characteristics obtained at lower contraction intensities would reflect the BB force–length characteristics better indicating the BB to operate mostly in the ascending limb of the force–length curve but cover a small portion of the descending limb at full elbow extension. To test these hypotheses, in the future, direct force measurements of the BB at the respective tendons or SWE measurements of multiple muscles involved in elbow flexion could be performed. These would complement our present findings and provide valuable information about the in vivo BB muscle force–length characteristics in both healthy and diseased condition.

### 4.2. MG Patients Show Increased Muscle Activity during Rest

Clinical electrophysiological examinations for neuromuscular junction disorders such as MG include the tests for repetitive nerve stimulations and neuromuscular jitter [35]. However, muscles are not evaluated in their passive state. This is the first study to report the sEMG amplitude of MG patients during rest, showing an average muscle activity of more than 5% in the relaxed condition. Considering a threshold of 3% muscle activity as a criterion of passive muscle state, we conclude that resting state of the MG patients with increased muscle activity does not represent their passive muscle state. To measure the passive state of a muscle in its in vivo environment, participants are asked to relax their muscle as much as possible. This requires the ability to voluntarily relax a muscle, which might be altered with MG. In spite of that, an increased sEMG amplitude during rest can be explained in two ways: (i) there is indeed an increased muscle activity during rest for MG patients or (ii) the amplitude used for normalization is small, leading to a narrow margin from rest to maximum activity. sEMG amplitudes need a normalization to compare and interpret the data between participants and measured conditions and there exist different approaches for normalization, whereas using the amplitude measured during MVC is the most common [36]. As MG is characterized by a disturbed neuromuscular transmission and as a result muscle weakness, decreased electrical activity seems reasonable. However, comparing absolute sEMG amplitudes between participants is not feasible and therefore this explanation cannot be verified from sEMG amplitudes.

Independent of the underlying reason, electromechanical adaptation of muscle takes place in MG. A direct comparison of the passive shear elastic modulus of MG patients with the healthy population (Figure 5) confirms that the passive muscle mechanics are altered with MG. Considering similar boundary conditions for both groups in passive state, the higher shear wave speed can be attributed to higher muscle stiffness in MG. However, the present study does not consider the viscoelasticity of muscle tissue, which may also influence the measured shear wave speed [17]. Thereby, it remains an open question whether MG also alters viscosity in muscle tissue and it should be the interest of further studies to investigate the underlying mechanisms of the muscle adaptations (both in stiffness and rheological properties) in MG. Though, the present increase in passive muscle stiffness must be interpreted with caution due to the small sample size of MG patients and as the two groups are not age-matched. We know that muscle stiffness alters with age; however, the results are not consistent across different studies reporting increased [37,38] and decreased muscle stiffness [39,40] with age. Nevertheless, the increased sEMG activity at rest might be one of the main indicators of muscle weakness and the patients’ complaint of exhaustion in MG. The negative correlation between the maximum elbow torque and sEMG amplitude and shear elastic modulus during rest support this hypothesis as it indicates that patients with increased muscle activity during rest could generate lower torque.

### 4.3. Detecting Muscle Weakness in MG Using SWE

Within this cohort of MG patients, we found considerable muscle weakness due to MG: during MVC elbow flexion, MG patients performed 73.54% of the elbow torque of the healthy group, indicating a reduced force production capability of the BB and other upper arm muscles involved. Only the active shear elastic modulus reflected the lower torque by a lower muscle stiffness but not the total shear elastic modulus. On the other hand, during submaximal contractions, (i) the total shear elastic modulus of MG patients compared to the healthy group was lower at a high contraction intensity (75% MVC) and (ii) for the active shear elastic modulus at all contraction intensities. We assume that the higher muscle stiffness of MG patients measured during rest shadows the lower muscle stiffness imposed by the reduced active force production capability, in particular for low contraction intensities. At higher contraction intensities, the effect of the resting shear elastic modulus is not big enough to shadow the increasing muscle stiffness resulting from force production and therefore the total shear elastic modulus is lower for MG patients. Following this explanation, we expect the total shear elastic modulus during MVC to be lower for MG patients. However, this was not the case. Interestingly, the muscle stiffness during MVC was found to be similar or even lower than the muscle stiffness at 75% MVC for both healthy and MG patients, indicating that not only contraction intensity but also contraction type (i.e., fast contraction vs. slow contraction) may affect the muscle stiffness.

To conclude, from both maximal and submaximal contraction intensities, our results suggest that the lower active shear elastic modulus reflects the muscle weakness of MG patients. Considering that the measured shear wave speed reflects the force-dependent muscle stiffness (which is positively correlated to the force production) and also the muscle tension [16,32], a lower shear wave speed in MG indicates lower force production (muscle weakness). Though the individual impact of these two factors should further be investigated. Nevertheless, the present results are promising that with further study of SWE in active muscle state, muscle weakness can be described by SWE.

### 4.4. Implications, Limitations and Clinical Use

Despite the aforementioned challenges in the interpretation of the measured shear wave speed from SWE, the present study revealed the potential of SWE to characterize diseased muscle in MG by demonstrating an increased muscle stiffness in MG patients during rest. Moreover, we found lower shear wave speeds in the active state and argued that these can be ascribed to the patients’ muscle weakness. Hence, SWE is promising to be used as a quantitative, objective diagnostical tool in MG. However, it should be noted that muscle stiffness (shear elastic modulus) was calculated from the shear wave speed assuming transverse isotropic and lossless linear elastic material characteristics that might not represent the true material behavior, and the influence of tensile loading on shear wave speed especially during active state needs to be addressed in future.

Recruiting patients with a rare disease is challenging. Even though the sample size of 11 was enough to provide statistically meaningful results, it did not allow us to analyze patients in subgroups based on age, sex and severity of MG (as reflected by, e.g., regular sport activities and the Besinger Score). These should be the reason for observed inter-individual variance in maximum torque production. A bigger sample size would allow more detailed categorization while presumably increasing the heterogeneity even more. Despite the high inter-individual variance, SWE was able to reflect the changes in passive muscle force imposed by different joint positions and the active muscle force differences imposed by different contraction intensities. Thus, we demonstrated that SWE can be used to mechanically characterize muscles of MG patients. As ultrasound elastography measurements are non-invasive and risk-free, there is no contraindication to it being used in the clinical routine.

Moreover, the increased sEMG activity and increased muscle stiffness during rest might be two main signs of muscle weakness in MG. By investigating a larger cohort of MG patients with heterogenous muscle weakness, this hypothesis can be further tested and, if validated, has great potential to be used in clinical diagnostics.

In conclusion, SWE seems to be a promising tool to characterize muscles affected by MG in both passive and active state and to objectively describe muscle weakness in MG. SWE measurements can give additional insights about a patient’s condition not only for MG but also for other neuromuscular diseases. Using SWE as a prospective index of muscle passive and active forces as well as to characterize muscle adaptation is valuable for monitoring neuromuscular diseases and deciding on the treatment options.

## Figures and Tables

**Table 1:** Patient characteristics.

ID	Sex	Age	BMI (kg/m^2^)	Handedness	Antibody Specificity	Medicaments *	Regular Sports	B.S., Total	B.S., Arms
1	F	64	23.88	Left	AChR	Mycophenolatmofetil 1500 mg	-	2	0
2	M	42	29.83	Right	AChR	Azathioprin 150 mg, Pyridostigmin ret. 720 mg	Running, volleyball	0	0
3	M	23	25.08	Right	MuSK	Pyridostigmin 90 mg	Bouldering	1	0
4	F	57	33.62	Right	AChR	Rituximab	Walks	2	0
5	F	35	24.68	Right	AChR	Prednisolon 3 mg, Immunoglobulins, Pyridostigmin ret. 270 mg	Aqua jogging	5	1
6	M	24	23.45	Right	CMS	Pyridostigmin 180 mg + ret. 90 mg	Walks	7	2
7	F	60	30.00	Right	AChR	Prednisolon 7.5 mg, Pyridostigmin 180 mg	-	5	1
8	F	65	32.32	Right	AChR	Mycophenolatmofetil 750 mg, Pyridostigmin 60 mg + ret. 90 mg	-	6	2
9	M	53	22.13	Right	MuSK	Rituximab	Biking, walks, gym	5	1
10	F	62	22.43	Right	MuSK	Rituximab	Yoga, Walks	9	1
11	F	39	23.23	Right	AChR, Anti-Titin	Azathioprin 175 mg, Pyridostigmin 180 mg + ret. 90 mg	Walks	7	0

BMI: Body mass index; * Daily dosage with Pyridostigmin as symptomatic therapy separated in retarded (ret.) and not retarded; B.S.: Besinger Score; AChR: Acetylcholine receptors; MuSK: Muscle-specific receptor tyrosine kinase; CMS: Congenital myasthenic syndrome with AChR receptor deficit.

**Table 2:** Experimental protocol.

Step	Trial Type	Duration	Elbow Angles
1	Extension MVC	5 s	120°
	Break	2 min	
2	Rest	5 s	60–180°
3	Flexion MVC	5 s	60–180°
	Break	2 min	
4	Ramp contraction up to 25% MVC torque	19 s	60–180°
	Break	1 min	
5	Ramp contraction up to 50% MVC torque	27 s	60–180°
	Break	1 min	
6	Ramp contraction up to 75% MVC torque	35 s	60–180°
	Break	1 min	

MVC: Maximum voluntary contraction.

**Table 3:** Individual maximum elbow torque during maximum voluntary contraction (MVC) in flexion and extension directions.

	MVC Torque (Nm)					
Patient ID	Elbow Angles: Flexion					Extension
	60°	90°	120°	150°	180°	120°
1	27.91	21.32	17.92	4.60	8.63	13.61
2	51.15	54.35	47.63	36.24	27.95	38.64
3	76.02	72.47	74.32	54.65	43.08	29.21
4	18.90	20.53	22.11	16.51	8.84	14.14
5	25.04	26.21	28.77	23.29	15.70	18.36
6	44.49	36.06	36.25	31.53	37.90	24.53
7	24.72	29.83	19.12	19.04	19.12	16.33
8	8.90	10.68	14.10	9.05	6.94	7.97
9	57.19	55.64	48.26	37.81	41.12	26.61
10	14.77	21.53	17.12	12.33	9.06	16.33
11	25.99	19.35	18.86	21.60	8.50	13.38
Mean	35.35	33.45	31.32	24.24	20.62	20.17
STD	18.67	18.33	17.84	14.01	13.66	8.29

MVC: Maximum voluntary contraction; STD: Standard deviation.

**Table 4:** Pearson correlation test findings for the maximum voluntary elbow flexion torque.

		BB CSA		SWE at MVC		sEMG at Rest		SWE at Rest	
		R Value	*p* Value	R Value	*p* Value	R Value	*p* Value	R Value	*p* Value
**MVC elbow flexion torque**	60°	**0.642**	**0.033**	**0.914**	**<0.001**	**−0.625**	**0.040**	**−0.670**	**0.024**
	90°	0.583	0.060	**0.869**	**0.001**	−0.335	0.314	−0.574	0.065
	120°	0.515	0.105	0.285	0.395	−0.427	0.181	−0.458	0.157
	150°	0.599	0.052	0.017	0.959	−0.726	0.155	−0.444	0.172
	180°	**0.778**	**0.005**	0.223	0.510	**−0.601**	**0.022**	**−0.682**	**0.021**

MVC: Maximum voluntary contraction; BB: Biceps brachii muscle; CSA: Cross-sectional area; SWE: Shear wave elastography; sEMG: Surface electromyography; significant results are highlighted in bold.

**Table 5:** Pearson correlation test findings for the surface electromyography measured at rest.

		SWE at Rest		Age		BMI		BB CSA	
		R Value	*p* Value	R Value	*p* Value	R Value	*p* Value	R Value	*p* Value
**sEMG at rest**	60°	**0.941**	**<0.001**	0.465	0.150	**0.647**	**0.031**	−0.276	0.411
	90°	**0.763**	**0.006**	0.481	0.134	0.572	0.066	−0.243	0.472
	120°	0.516	0.105	**0.693**	**0.018**	0.389	0.237	−0.580	0.061
	150°	**0.808**	**0.003**	0.567	0.069	0.479	0.136	−0.444	0.171
	180°	0.432	0.184	0.354	0.285	0.250	0.458	−0.355	0.285

sEMG: Surface electromyography; SWE: Shear wave elastography; BMI: Body mass index; BB: Biceps brachii muscle; CSA: Cross-sectional area; significant results are highlighted in bold.

## Data Availability

The data presented in this study are available on request from the corresponding author.
